# A case report of psychiatric symptoms following direct-acting antiviral and ribavirin combination therapy for chronic hepatitis C in a patient with innate anxiety

**DOI:** 10.1186/s12876-019-1013-1

**Published:** 2019-06-13

**Authors:** Akira Sakamaki, Kenya Kamimura, Naoki Fukui, Haruka Watanabe, Norihiro Sakai, Kentaro Tominaga, Kenichi Mizuno, Masaaki Takamura, Hirokazu Kawai, Takuro Sugai, Satoshi Yamagiwa, Toshiyuki Someya, Shuji Terai

**Affiliations:** 1 Division of Gastroenterology and Hepatology, Graduate School of Medical and Dental Sciences, Niigata University, 1-757 Asahimachidori, Chuo-ku, Niigata, 951-8510 Japan; 2 Division of Psychiatry, Graduate School of Medical and Dental Sciences, Niigata University, 1-757 Asahimachidori, Chuo-ku, Niigata, 951-8510 Japan

**Keywords:** Interferon, Direct-acting antiviral, Psychiatric symptom, Case report

## Abstract

**Background:**

Direct-acting antivirals (DAAs) result in a highly sustained virological response rate and better patient tolerance. However, this therapeutic approach may, on rare occasions, give rise to psychiatric symptoms. We describe a case requiring discontinuation of DAA and ribavirin combination therapy due to psychiatric symptoms in a patient with congenital anxious personality traits. The information summarized here will be helpful to physicians treating chronic hepatitis C virus (HCV) infection in patients with underlying psychiatric problems.

**Case presentation:**

A 57-year-old Japanese woman diagnosed with chronic HCV infection was prescribed DAA and ribavirin combination therapy. She had a history of mild innate anxiety and development of psychiatric symptoms due to interferon (IFN) therapy 8 years prior, which subsided with discontinuation of the therapy. Similar psychiatric symptoms such as enervation, palpitations, an episode of hyperventilation, and consciousness disturbances with myotonia were observed after the administration of the antiviral agents. No abnormal findings related to her symptoms were observed on laboratory or imaging results. Psychiatrists diagnosed the patient as having a somatization disorder induced by the antiviral agents on the basis of innate anxiety. After the discontinuation of therapy, her symptoms gradually improved.

**Conclusions:**

Although DAAs were not causative factors for psychiatric symptoms in phase 3 studies, a post-marketing study reported psychiatric symptoms such as depression in patients with underlying psychiatric problems. Our case suggests psychiatric symptoms might worsen after DAA and ribavirin administration in patients with underlying psychiatric disorders, and therefore, close monitoring is necessary for these patients, especially if they have a history of psychiatric symptoms after IFN.

## Background

Chronic hepatitis C virus (HCV) infection is one of the main causes of chronic liver disease, and approximately 2.2% of adults worldwide are infected [1]. In addition, 10–15% of these cases progress to liver cirrhosis and hepatocellular carcinoma, leading to a fatal outcome [2–4]. The first established therapy for hepatitis C was interferon (IFN) reported by Hoofnagle et al. in 1986, wherein administration of human recombinant IFN-α resulted in normalization of transaminases in patients with non-A, non-B hepatitis [5]. IFN-α monotherapy (thrice weekly, 6 million international units per day) resulted in sustained virological response (SVR) of only 5% in resistant cases such as genotype 1 with high viral ribonucleic acid levels; therefore, the development of PEGylated IFN (PEG-IFN) and ribavirin combination therapy contributed to the increase in SVR rates (approximately 42–56%) [6, 7]. Thus, IFN and its combination therapies significantly decreased the occurrence of hepatocellular carcinoma and improved prognoses [8]. Conversely, in some cases, PEG-IFN and ribavirin combination therapy induces various adverse events including cytopenia, influenza-like, gastrointestinal, and psychiatric symptoms that may require discontinuation of the therapy [6, 7].

HCV treatment paradigms have dramatically shifted with the development of direct-acting antivirals (DAAs) that provide SVR rates as high as 80–100% and with better patient tolerance [9]. However for a portion of IFN-intolerant patients, psychiatric adverse effects have thwarted efforts using DAA therapy. In this report, we present the case of an innate anxiety patient with a history of IFN discontinuation due to psychiatric symptoms and who developed similar symptoms using DAA and ribavirin to treat her HCV hepatitis. The information summarized is intended for physician awareness of possible drawbacks of IFN-free treatment for HCV infection in patients with underlying psychiatric issues.

## Case presentation

**Fig. 1:**
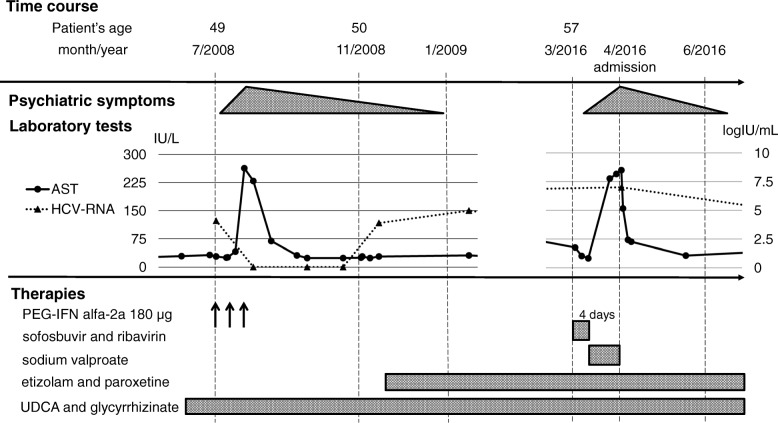
Clinical course of psychiatric symptoms, laboratory tests, and therapies. After subcutaneous PEG-IFNα-2a injection, the 49-year-old patient experienced psychiatric symptoms and therefore, treatment was discontinued; anti-anxiety medications were administered, and the symptoms gradually disappeared. At age 57, IFN-free therapy was initiated and 4 days after administration psychiatric symptoms were observed. Attending psychiatrists diagnosed somatization disorder, and antiviral drugs were discontinued, following which symptoms gradually decreased and finally disappeared. AST, aspartate aminotransferase; IU, international unit; HCV-RNA, quantitative hepatitis C virus ribonucleic acid; PEG-IFN, PEGylated interferon; UDCA, ursodeoxycholic acid; meshed pattern triangles, intensity of psychiatric symptoms; black arrows, subcutaneous injection; meshed pattern boxes, administration period of treatment agents

A 49-year-old Japanese woman was presented with chronic hepatitis due to HCV genotype 2 infection. She had a psychiatric history of mild innate anxiety but was not medicated. She was prescribed subcutaneous injection of PEG-IFNα-2a at a dose of 180 μg per week to treat chronic hepatitis. After initiation of therapy, a low-grade fever and mild general fatigue were observed. Psychiatric symptoms such as enervation, palpitations, an episode of hyperventilation, and consciousness disturbances with myotonia appeared after the third injection of PEG-IFNα-2a. It was impossible to decide if the symptoms were IFN-related or due to a somatization disorder elicited by anxiety, but the IFN therapy was discontinued and followed by administration of etizolam and paroxetine hydrochloride hydrate treatment by psychiatrists. Although the symptoms gradually improved, it took 3 months for the patient to completely recover (Fig. 1); the anti-anxiety medications were continued for a prolonged period. While waiting for approval of DAA therapy, the patient was administered liver supporting therapies: oral ursodeoxycholic acid and glycyrrhizinate. Upon approval, the patient was administrated with IFN-free sofosbuvir and ribavirin combination therapy. She was 57 years at this time point and 8 years had elapsed since the PEG-IFN therapy. The patient remained diagnosed with chronic hepatitis, as she showed a low score of 2.50 in the fibrosis-4 index [10] and aspartate aminotransferase-to-platelet ratio index [11] was 0.731. Unexpectedly, psychiatric symptoms similar to those observed with IFN and consciousness disturbance attacks appeared 4 days after treatment initiation. Initially, psychiatrists attributed the symptoms to epileptic seizures, and sodium valproate was administrated. However, her symptoms did not improve, and the patient was admitted for further observation and treatment.

**Fig. 2:**
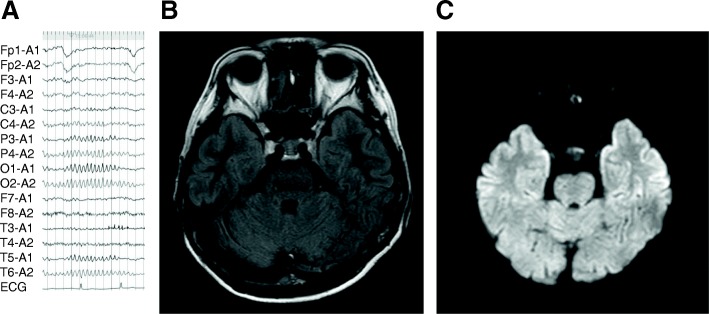
Imaging findings on admission with psychiatric symptoms after interferon-free therapy. Electroencephalogram showed 10–11 Hz with an irregular unsteady α wave dominant in the occipital lobe and without paroxysmal abnormality (**a**). Brain magnetic resonance imaging in fluid-attenuated inversion recovery (**b**) and diffusion-weighted imaging (**c**) revealed normal findings, and drug-induced encephalopathy was not developed

Physical examination was unremarkable. The liver and spleen were not palpable, and her bowel sounds were normal. Anemia and jaundice were not seen in palpebral conjunctiva or bulbar conjunctiva. Flapping tremor and leg edema were absent. Abnormal neurological finding were not detected. The patient denied alcohol and/or drug abuse. Upon admission, the patient was administrated etizolam and paroxetine for anxiety disorder, ursodeoxycholic acid and glycyrrhizinate for chronic hepatitis, and metoprolol and enalapril for chronic heart failure after a surgical operation for endocardial cushion defect. Other than a mild increase in serum aspartate and alanine aminotransferase levels due to sodium valproate administration, no abnormal laboratory findings, including ammonium or glycemic levels that might induce consciousness disturbances, were found (Table 1). There was no evidence for HBV and HIV co-infection. Furthermore, the patient’s electroencephalogram and brain magnetic resonance imaging findings were normal (Fig. 2a-c). These results indicated no evidence of infection or hepatic or drug-induced encephalopathy. In addition, the patient had stable vital signs and communicated well even during the psychiatric attacks unless prompted about hepatitis related topics (which would not have been the case if she was suffering from epileptic seizures). Based on the clinical picture, psychiatrists confirmed a diagnosis of somatization disorder induced by anxiety from antiviral therapy. Sodium valproate, sofosbuvir, and ribavirin were discontinued, and her symptoms gradually disappeared after 3 months. Anti-anxiety medication was continued for treatment of the somatization disorder; the patient continued to receive liver supporting therapies because of a mild increase in serum aspartate and alanine aminotransferase levels; interruption of antiviral therapy showed no clearance of HCV. With the combination of mental health support from psychiatrists, we are planning to retry an alternate DAA regimen without ribavirin.

**Table 1:** Laboratory data on admission with psychiatric symptoms after sofosbuvir and ribavirin combination IFN-free therapy

Hematology		Normal Value	Biochemistry		Normal Value
Leukocyte count (/mm^3^)	3370	3300–8600	Total Protein (g/dl)	7.2	6.6–8.1
Erythrocyte count (× 10^4^/mm^3^)	450	386–492	Albumin (g/dl)	**3.8**	4.1–5.1
Hemoglobin (g/dl)	13.7	11.6–14.8	Serum sodium (mEq/l)	140	138–145
Hematocrit (%)	42.3	35.1–44.4	Serum potassium(mEq/l)	3.8	3.6–4.8
Platelet count (× 10^4^/mm^3^)	**10.2**	15.8–34.8	Serum chloride(mEq/l)	104	101–108
Coagulation test			Serum IP (mg/dl)	3.1	2.5–4.5
			Serum calcium (mg/dl)	9.5	8.8–10.1
Prothrombin time (%)	93	70–130	Total bilirubin (mg/dl)	0.6	0.4–1.5
PT-INR	1.04	1.0	Direct bilirubin (mg/dl)	0.1	< 0.3
Tumor marker			AST(IU/l)	**255**	13–30
			ALT(IU/l)	**434**	7–23
α-fetoprotein (ng/ml)	3	< 9.5	LDH (IU/l)	**252**	124–222
AFP-L3(%)	< 0.5	< 10.0	ALP(IU/l)	**376**	106–322
Viral marker			GGT(IU/l)	**81**	9–32
			Blood urea nitrogen(mg/dl)	12	8–20
HCV-Ab	**positive**	negative	Creatinine (mg/dl)	0.60	0.46–0.79
HCV serotype	2		Fasting blood sugar (mg/dl)	132	
HCV-RNA (logIU/ml)	**7.0**	negative	Serum ammonia (μl/dl)	47	12–66
			C-reactive protein (mg/dl)	0.01	< 0.15
HBsAg	negative	negative	Thyroid hormone		
HIV-Ab	negative	negative			
			Free triiodothyronine (pg/ml)	3.5	2.3–4.0
			Free thyroxine (ng/dl)	1.3	0.9–1.7

Abnormal values are given in bold type

PT-INR, international normalized ratio of prothrombin time; AFP-L3, *Lens culinaris* agglutinin a-reactive α-fetoprotein; HCV-Ab, hepatitis C virus-antibody; HCV-RNA, quantitative hepatitis C virus-ribonucleic acid; HBsAg, hepatitis B surface antigen; HIV-Ab, human immunodeficiency virus-antibody; IP, inorganic phosphorus; AST, aspartate aminotransferase; ALT, alanine aminotransferase; LDH, lactate dehydrogenase; ALP, alkaline phosphatase; GGT, gamma-glutamyl transpeptidase

## Discussion and conclusions

A meta-analysis of HCV patients demonstrated evidence for high incidence rates of depression and/or anxiety even in untreated patients [12]. It is well known that IFN therapy precipitates psychiatric symptoms such as depression, irritability, anxiety, agitation, loss of appetite, fatigue, sleep disturbance, and impaired cognition; it is difficult to distinguish underlying symptoms from IFN-related ones [13]. Risk factors for IFN-related psychiatric symptoms include the following: high dose of IFN, older age, history of organic brain syndrome, current psychiatric diseases, or depression or insomnia, drug abuse, and disease or therapy anxiety [14]. IFN-related psychiatric adverse events lead to discontinuation of therapy or hospital admission in 10% of cases, whereas 30% of cases continue IFN therapy with supportive therapy such as anti-depressant or anti-anxiety medication [15, 16]. In several patients with psychiatric symptoms, intolerance to IFN resulted in the future of HCV elimination.

DAA therapy, on the other hand, is associated with high SVR rates and better patient tolerance, especially for psychiatric symptoms, as reported in a phase 3 study that excluded patients with underlying psychiatric disorders [9]. Ledipasvir and sofosbuvir combination therapy showed SVR rates of 99%, with no discontinuation owing to adverse events and no treatment-related depression [17]. Similarly, high SVR rate and fewer adverse events were reported with DAA combination therapy with ribavirin for HCV genotype 2 patients [18]. However, in a phase 4, open-label study, 6.1% of patients with liver cirrhosis, reported depression and an equal rate of insomnia in a patient population where 35% suffered from underlying depression, 30% from underlying anxiety or anxiety disorder, and 10% from alcohol abuse [19]. Takeda et al. reported a mild increase in depression in the first 4 weeks after administration of DAAs, which recovered within 12 weeks [20], as revealed by the Beck Depression Inventory scores, which are calculated by a 21-question multiple-choice self-application instrument to assess the severity of depression [21]. The authors suggested that anxiety associated with antiviral treatment might worsen depression scores, regardless of the regimen, which is consistent with the clinical course observed in our case. Although, to our knowledge, DAAs do not directly affect the central nervous system, Volpato et al. reported DAAs may have mild neurotoxicity in patients with LC, because of increased relative delta power in the electroencephalogram at the end of treatment with DAAs [22]. Conversely, Sundberg et al. reported no increase in depression scores on other self-rating scales after DAA therapy [23]. Further studies are required to determine whether DAAs affect the central nervous system and manifest psychiatric symptoms, since patients with underlying psychiatric disorders are sensitive to psychiatric adverse events associated with DAA therapy.

Furthermore, ribavirin may worsen the psychiatric symptoms. Ribavirin combination therapy with both PEG-IFN [6] and DAA [24] significantly increased not only anemia, but also psychiatric symptoms such as fatigue and insomnia compared with PEG-IFN or DAA monotherapy.

In conclusion, psychiatric disease is common among patients with chronic HCV infection, and those undergoing IFN-based treatment, but our case signals the possibility of psychiatric effects with oral DAA regimens as well. Therefore, close monitoring is necessary for patients who have psychiatric risk factors similar to IFN-related psychiatric symptoms, such as history of depression, insomnia, or anxiety to disease or medication, and who are undergoing IFN-free therapy, especially ribavirin combination regimens.

## Data Availability

All data generated or analysed during this study are included in this published article.

## Abbreviations

DAA: Direct-acting antiviral
HCV: Hepatitis C virus
IFN: Interferon
PEG-IFN: PEGylated interferon
SVR: Sustained virological response
